# Supplementary material for: Hippocampal mechanisms underlying the resolution of competition in memory and perception
Source: Nat Commun. 2026 Sep 4;17:10528. doi: 10.1038/s41467-026-77400-8 (PMC13639053; doi:10.1038/s41467-026-77400-8)
Supplement: Supplementary file 1 [file 41467_2026_77400_MOESM1_ESM.pdf]

Supplementary Information

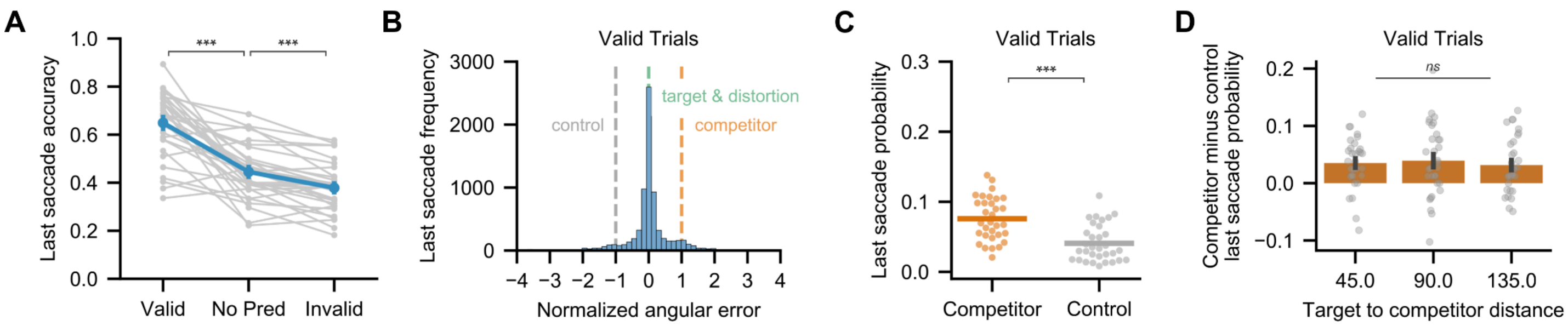

Supplementary Figure 1. Last saccade behavioral data from memory-guided visual search task (session 2). (A) Relative to no-prediction trials, participants' last saccade was more accurate on valid trials ($t(31) = 7.34$, $p < 0.001$, $d_z = 1.62$, valid - no-prediction = 0.20, 95% CI = [0.15, 0.26]) and less accurate on invalid trials ($t(31) = -5.47$, $p < 0.001$, $d_z = 0.62$, invalid - no-prediction = -0.067, 95% CI = [-0.09, -0.04]), demonstrating memory-guided search (N = 32 participants, two-tailed paired t-tests). Gray lines indicate individual participants; blue lines indicate across-participant mean ± SEM. **(B)** Last saccade angular error distribution on valid trials. Errors are normalized by target-competitor distance (x=0: target/distortion; x=1: competitor; x=−1: equidistant control). Saccades were most frequent near the target, but the distribution is asymmetric toward the competitor. **(C)** Last saccade probabilities for competitor and control locations from (B). Saccades to the competitor were more frequent than to the control ($t(31) = 5.05$, $p < 0.001$, $d_z = 1.22$, competitor - control = 0.035; 95% CI = [0.02, 0.05]; N = 32 participants, two-tailed paired t-test), suggesting that competition persisted into the search task. Dots indicate individual participants; lines indicate across-participant means. **(D)** Target-competitor distance did not significantly modulate last saccade likelihood to the competitor relative to control ($F(2,62) = 0.265$, $p = 0.768$, $\eta_p^2 = 0.008$; N = 32 participants; repeated measures ANOVA). Bars indicate across-participant mean ± SEM. ns = $p > 0.05$; *** $p < 0.001$

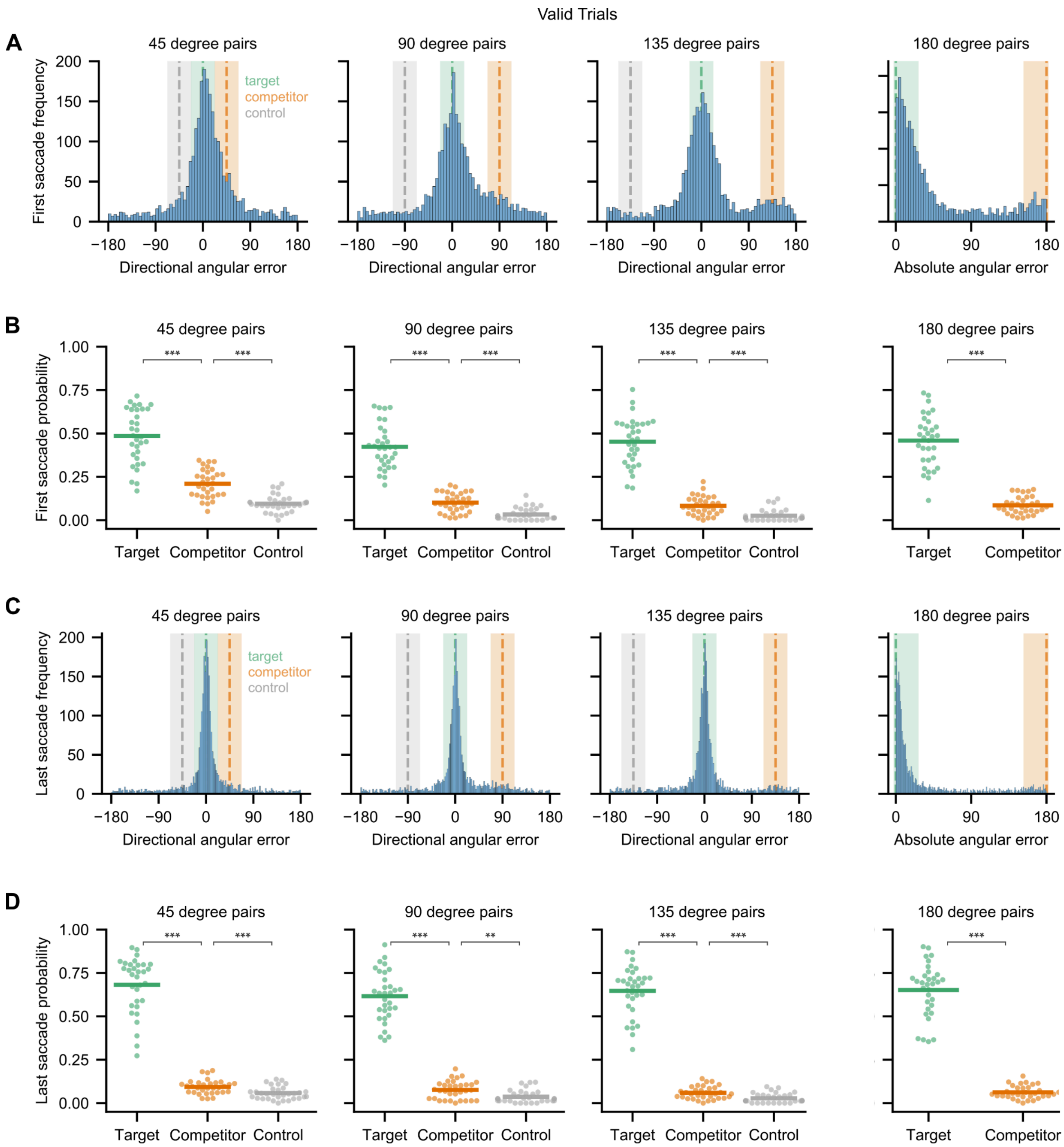

Valid Trials
A
B
C
D
45 degree pairs
90 degree pairs
135 degree pairs
180 degree pairs
target
competitor
control
First saccade frequency
Last saccade frequency
Directional angular error
Absolute angular error
First saccade probability
Last saccade probability
Target
Competitor
Control
***
**

Supplementary Figure 2. Behavioral data from memory-guided visual search task (session 2) separated by target-competitor distance. (A) The distribution of first saccade angular errors on valid trials, separately for each target-competitor distance. Zero degrees of error indicates a saccade made directly towards the target. For target-competitor distances of 45 degrees, 90 degrees, and 135 degrees, angular error is directional, such that positive values are toward the competitor and negative values are in the opposite direction toward a control location an equal distance away from the target as the competitor. For 180 degree pairs, no control location can be defined, so angular errors are collapsed across direction. Highlighted green areas represent the saccades that are identified to be at the target location. Orange and gray highlighted areas represent the saccades that are identified to be at the competitor and control locations, respectively. See Figure 2D for data normalized such that all target and competitor distances can be visualized at the same time. **(B)** Data from the target, competitor, and control shaded zones in (A) are re-plotted as probabilities for each participant. For every target-competitor distance, first saccades are made more often to the target than the competitor location. For the distances for which a control location can be defined, first saccades are made more often to the competitor than the control location. Dots represent individual participants and lines represent the across-participant mean. **(C)** Same as (A) but for the last saccade. **(D)** Same as (B) but for the last saccade. ** $p < 0.01$; *** $p < 0.001$

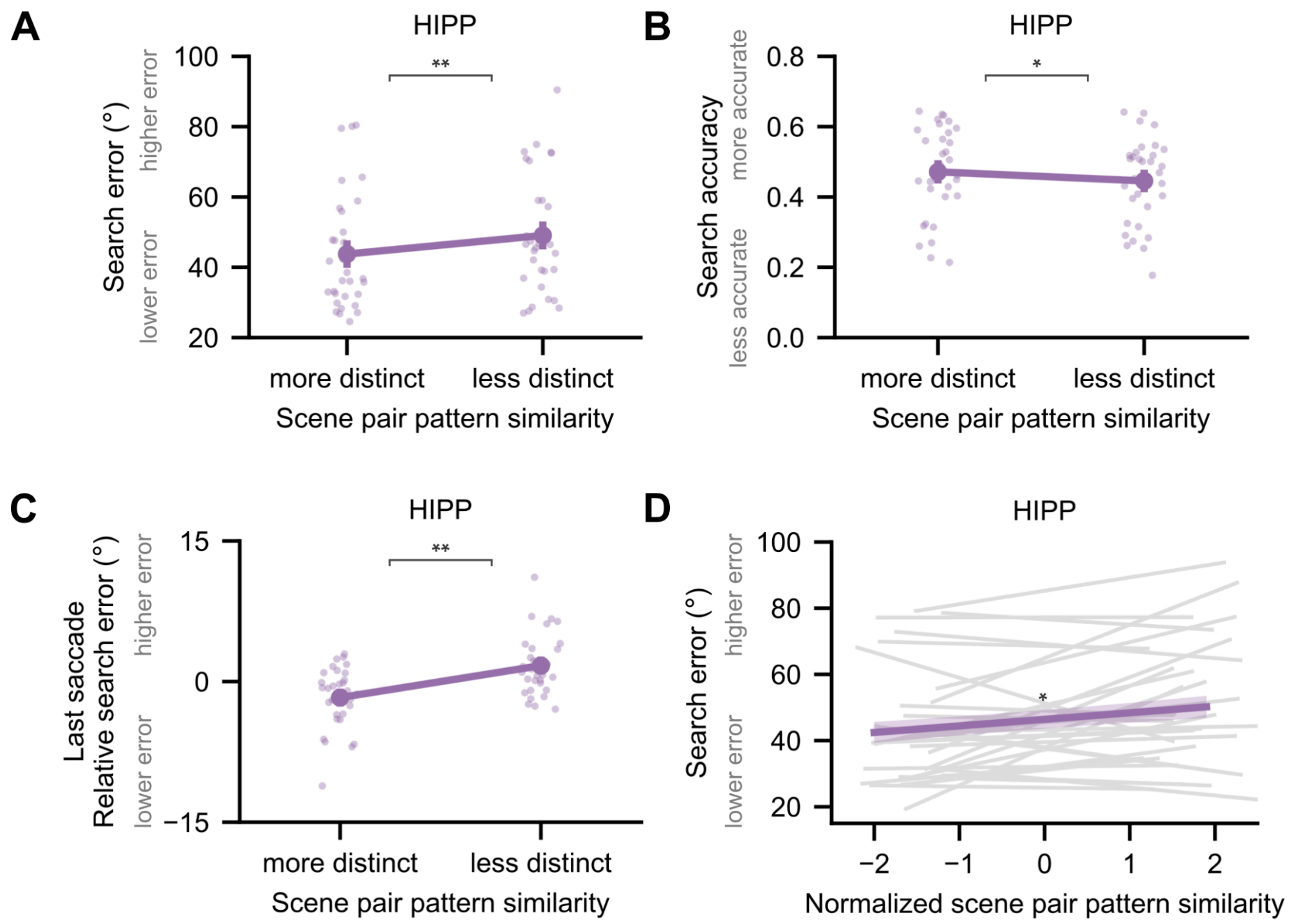

Supplementary Figure 3. Distinct hippocampal representations of competing scenes predict multiple measures of memory-guided eye movements. (A) The relationship between pattern distinctiveness in the hippocampus (HIPP) and search error on the next trial (t(31) = -3.46, p = 0.002, d_z = 0.32, more distinct - less distinct = -5.3 degrees, 95% CI = [-8.43, -2.18]; N = 32 participants, two-tailed paired t-test), visualized without centering data within participants. See Figure 3C for the same search error values centered within participants. **(B)** The relationship between pattern distinctiveness in the hippocampus and behavior was robust to binarizing search error into search accuracy (t(31) = 2.04, p = 0.049, d_z = 0.21, more distinct - less distinct = 0.026, 95% CI = [0.0, 0.05]; N = 32 participants, two-tailed paired t-test). Search accuracy was defined as the proportion of trials with first saccade errors less than 22.5 degrees. **(C)** The relationship between pattern distinctiveness in the hippocampus and behavior was robust when considering search error on the last saccade rather than the first saccade (t(31) = -3.01, p = 0.005, d_z = 0.23, more distinct - less distinct = -3.4 degrees, 95% CI = [-5.73, -1.10]; N = 32 participants, two-tailed paired t-test). For panels A-C, small dots represent individual participants. Large points represent the across-participant mean ± SEM. **(D)** The relationship between pattern distinctiveness in the hippocampus and behavior was robust when considering pattern distinctiveness as a continuous rather than binary measure (t(315) = 2.38, p = 0.018, β = 1.98, 95% CI = [0.34, 3.62]; N = 384 scene pairs across 32 participants; two-tailed post-hoc contrast on a linear mixed effects model). The purple line and shading represent the slope and standard error of the fixed effect from a model predicting search error from scene pair pattern similarity (normalized within participants). Gray lines represent the best fitting line to each participant's data. * p < 0.05; ** p < 0.01

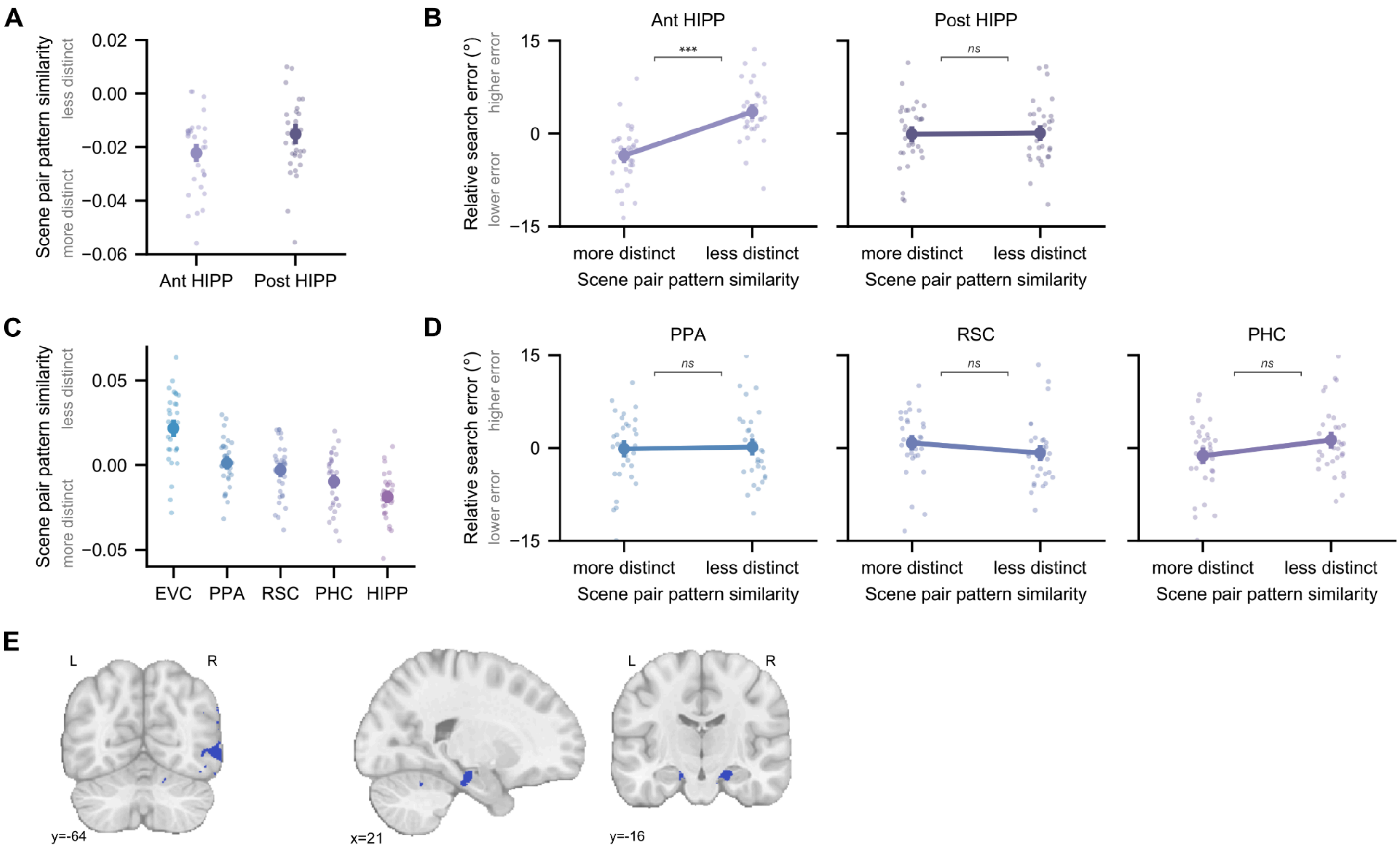

Supplementary Figure 4. Relationship between representations of competing scenes and memory-guided eye movements in other brain regions. (A) Average scene pair pattern similarity in anterior hippocampus (Ant HIPP) and posterior hippocampus (Post HIPP). **(B)** Scene pair pattern similarity in the anterior hippocampus, but not posterior hippocampus, predicted lower error saccades (anterior hippocampus: $t(31) = -4.33$, $p < 0.001$, $d_z = 0.43$, more distinct - less distinct = -7.1 degrees, 95% CI = [-10.44, -3.75]; posterior hippocampus: $t(31) = -0.11$, $p = 0.915$, $d_z = 0.01$, more distinct - less distinct = -0.19 degrees, 95% CI = [-3.84, 3.45]; N = 32 participants, two-tailed paired t-tests). **(C)** Average scene pair pattern similarity in early visual cortex (EVC) and hippocampus (as reported in the main text), parahippocampal place area (PPA), retrosplenial cortex (RSC), and parahippocampal cortex (PHC). **(D)** Scene pair pattern similarity did not significantly predict saccade error in PPA ($t(31) = -0.14$, $p = 0.890$, $d_z = 0.016$, more distinct - less distinct = -0.28 degrees, 95% CI = [-4.36, 3.80]), RSC ($t(31) = 0.90$, $p = 0.373$, $d_z = 0.098$, more distinct - less distinct = 1.63 degrees, 95% CI = [-2.05, 5.31]), or PHC ($t(31) = -1.32$, $p = 0.198$, $d_z = 0.15$, more distinct - less distinct = -2.55 degrees, 95% CI = [-6.51, 1.41]; N = 32 participants, two-tailed paired t-tests). For A-D, small dots represent individual participants. Large points represent the across-participant mean ± SEM. **(E)** Whole-brain searchlight clusters where lower scene pair pattern similarity (more distinct representations) was associated with lower search error on valid trials. Clusters are visualized for exploratory purposes at an uncorrected p value threshold of 0.01. Clusters were found in the right lateral occipital lobe (left) and the right hippocampus (right). ns = $p > 0.05$; ** $p < 0.01$

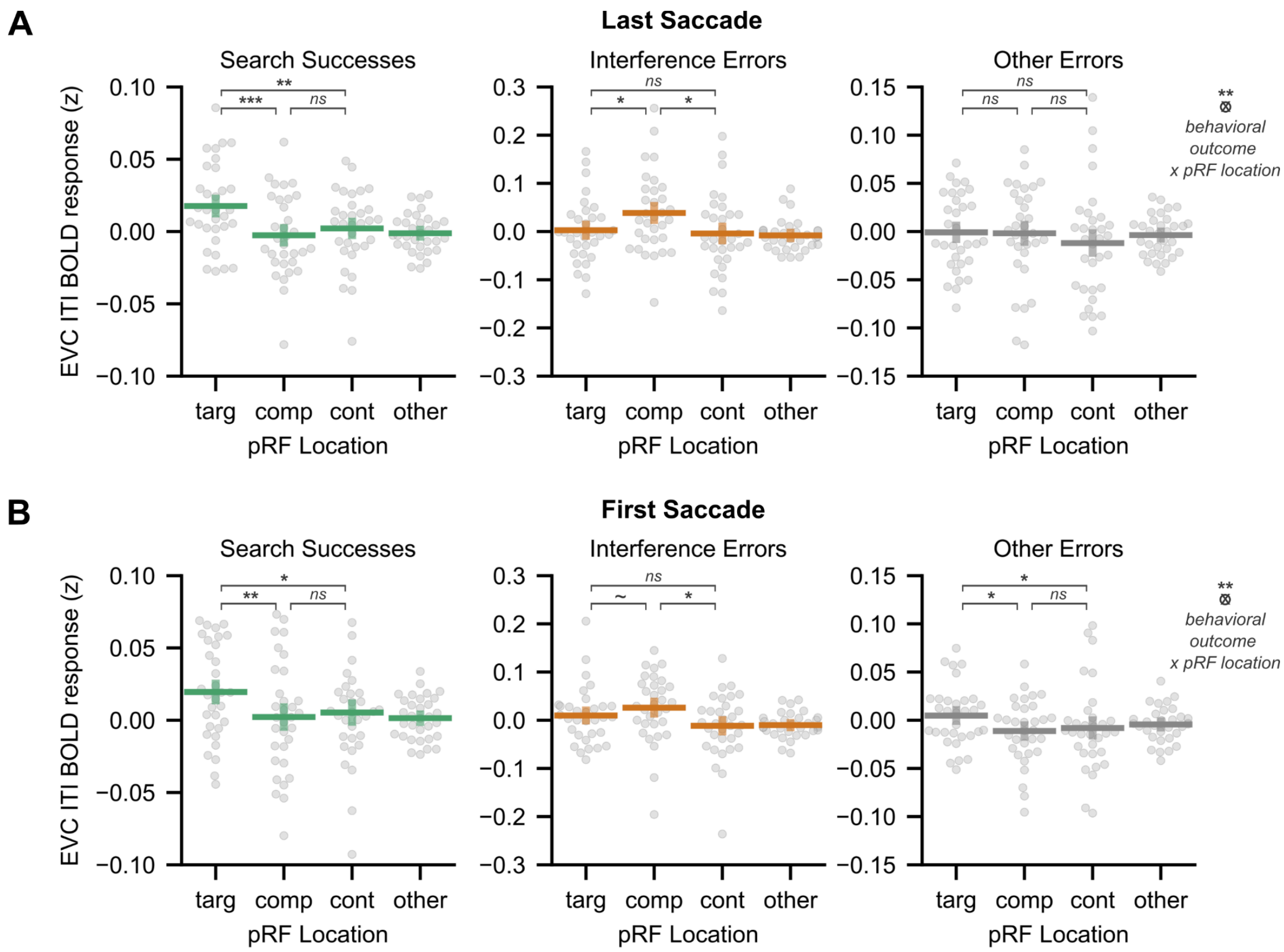

Supplementary Figure 5. Preparatory coding in early visual cortex as a function of behavioral outcome defined by the last and first saccade of search. We directly compared early visual cortex (EVC) BOLD activity during the intertrial interval (ITI) at population receptive fields (pRFs) corresponding to the target location (targ), competitor location (comp), control location (cont), and all other locations (other) for three behavioral outcomes: search successes, interference errors, and other errors. **(A)** We defined search outcomes according to the participant's last saccade of the search interval. Data from Figure 4D are replotted to visualize individual participants. **(B)** We defined search outcomes according to the participant's first saccade of the search interval. Note that because outcomes are defined here with respect to the first saccade, trials may ultimately end in successful search even if the first saccade was an error. A linear mixed-effects model revealed an interaction between pRF location and behavioral outcome ($F(6,75101) = 3.03$, $p = 0.006$; $N = 75144$ trials across 32 participants; Type III F-test). This interaction was driven by preferential target activation for search successes, weak preferential competitor activation for interference errors ($p = 0.112$), and preferential target activation for other errors. Differences between this figure and panel A, notably in the other errors condition, are due to saccades that were initially errors but were ultimately corrected. Only pairwise statistical comparisons between target, competitor, and control locations are shown. For both panels, small dots represent individual participants. Horizontal lines represent the across-participant mean and vertical lines represent the SEM. ns = $p > 0.05$; *$p < 0.05$; **$p < 0.01$; *** $p < 0.001$

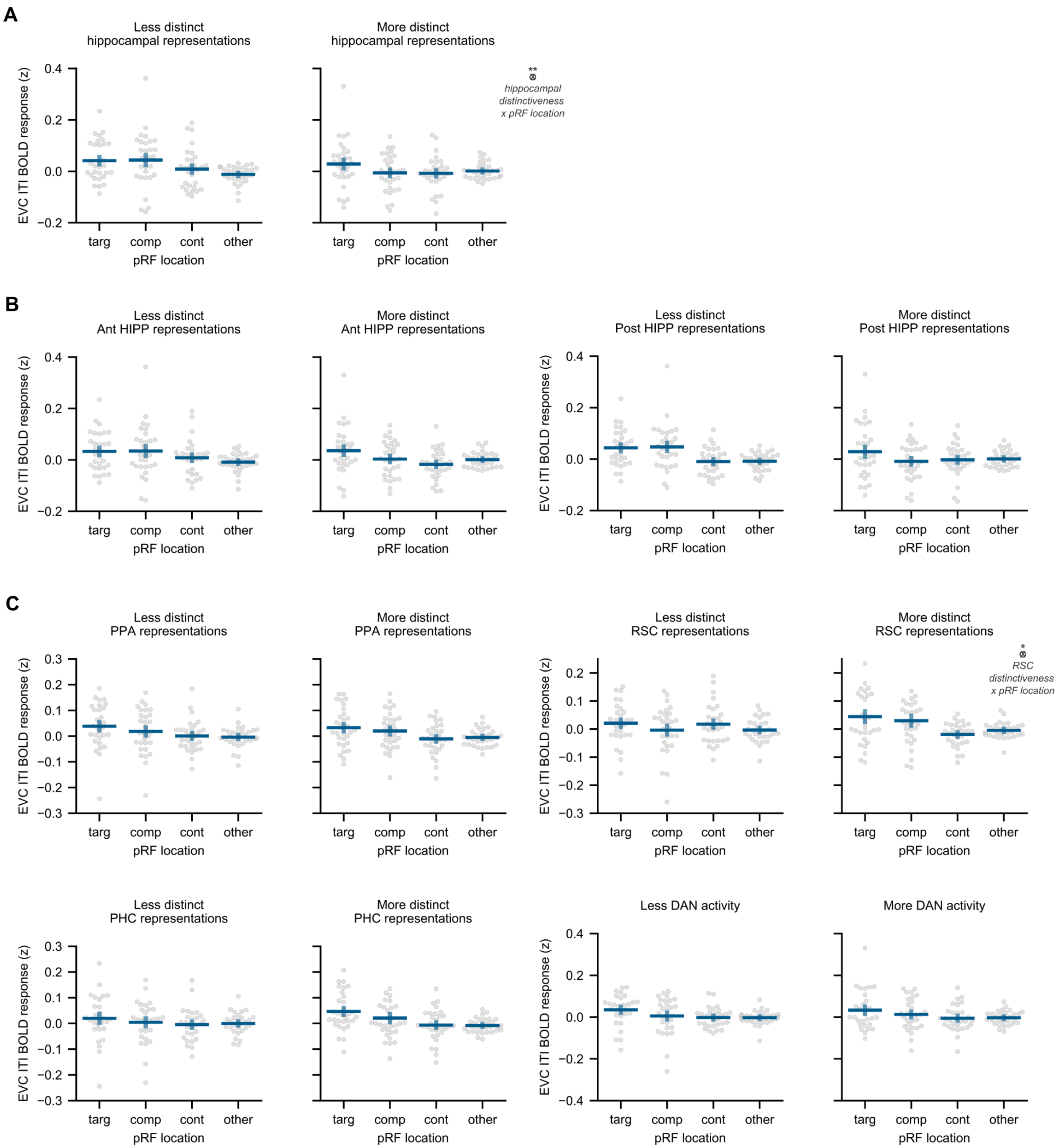
A
Less distinct hippocampal representations
More distinct hippocampal representations
** hippocampal distinctiveness x pRF location
EVC ITI BOLD response (z)
targ comp cont other
pRF location
B
Less distinct Ant HIPP representations
More distinct Ant HIPP representations
Less distinct Post HIPP representations
More distinct Post HIPP representations
C
Less distinct PPA representations
More distinct PPA representations
Less distinct RSC representations
More distinct RSC representations
* RSC distinctiveness x pRF location
Less distinct PHC representations
More distinct PHC representations
Less DAN activity
More DAN activity

Supplementary Figure 6. Brain regions outside the hippocampus are not associated with differences between target and competitor activation in visual cortex. (A) Data from Figure 5C is replotted to visualize individual participants. We repeated the analyses in Figure 5C in several brain regions outside the hippocampus. **(B)** We assessed scene distinctiveness in the anterior hippocampus (Ant HIPP) and posterior hippocampus (Post HIPP) separately. There was no significant interaction between hippocampal long-axis position, scene pair distinctiveness, and early visual cortex (EVC) population receptive field (pRF) location ($F(3,39236) = 1.12$, $p = 0.340$). We observed a qualitatively similar relationship in the anterior ($F(3,19588.8) = 2.39$, $p = 0.066$) and posterior hippocampus ($F(3,19591.4) = 2.29$, $p = 0.077$) as in the whole hippocampus: comparable target and competitor activation when scene representations were less distinct and higher target than competitor activation when scene representations were more distinct (Fig. 5C). **(C)** We assessed the effect of scene distinctiveness in parahippocampal place area (PPA), retrosplenial cortex (RSC), and parahippocampal cortex (PHC). For PPA and PHC, there was no significant interaction between EVC pRF location and PPA/PHC pattern distinctiveness (PPA: $F(3,19617) = 0.44$, $p = 0.725$; PHC: $F(3,19587.5) = 1.40$, $p = 0.239$). In RSC, there was an interaction ($F(3,19587.7) = 3.17$, $p = 0.023$), but the pattern of results was unlike those found in the hippocampus. Finally, we assessed the effect of univariate activity in the dorsal attention network (DAN). There was no significant interaction between EVC pRF location and univariate DAN activity ($F(3,19592.3) = 1.155$, $p = 0.325$). Note that we focus on the interaction with scene distinctiveness or univariate activity because main effects of target/competitor/control/other locations are not of primary interest. All statistics are Type III F-test run on N = 19656 trials across 32 participants. For all panels, small dots represent individual participants. Horizontal lines represent the across-participant mean and vertical lines represent the SEM. $^*p < 0.05$
