# Supplementary material for: Hippocampal mechanisms underlying the resolution of competition in memory and perception
Source: Nat Commun. 2026 Sep 4;17:10528. doi: 10.1038/s41467-026-77400-8 (PMC13639053; doi:10.1038/s41467-026-77400-8)
Supplement: Supplementary file 2 [file 41467_2026_77400_MOESM2_ESM.pdf]

nature portfolio

Corresponding author(s): Serra Favila

Last updated by author(s): June 17, 2026

Reporting Summary

Nature Portfolio wishes to improve the reproducibility of the work that we publish. This form provides structure for consistency and transparency in reporting. For further information on Nature Portfolio policies, see our Editorial Policies and the Editorial Policy Checklist.

Statistics

For all statistical analyses, confirm that the following items are present in the figure legend, table legend, main text, or Methods section.

n/a	Confirmed	
☐	☒	The exact sample size (*n*) for each experimental group/condition, given as a discrete number and unit of measurement
☐	☒	A statement on whether measurements were taken from distinct samples or whether the same sample was measured repeatedly
☐	☒	The statistical test(s) used AND whether they are one- or two-sided<br>*Only common tests should be described solely by name; describe more complex techniques in the Methods section.*
☒	☐	A description of all covariates tested
☒	☐	A description of any assumptions or corrections, such as tests of normality and adjustment for multiple comparisons
☐	☒	A full description of the statistical parameters including central tendency (e.g. means) or other basic estimates (e.g. regression coefficient) AND variation (e.g. standard deviation) or associated estimates of uncertainty (e.g. confidence intervals)
☐	☒	For null hypothesis testing, the test statistic (e.g. F, t, r) with confidence intervals, effect sizes, degrees of freedom and P value noted<br>*Give P values as exact values whenever suitable.*
☒	☐	For Bayesian analysis, information on the choice of priors and Markov chain Monte Carlo settings
☒	☐	For hierarchical and complex designs, identification of the appropriate level for tests and full reporting of outcomes
☐	☒	Estimates of effect sizes (e.g. Cohen's *d*, Pearson's *r*), indicating how they were calculated

Our web collection on statistics for biologists contains articles on many of the points above.

Software and code

Policy information about availability of computer code

Data collection	Data were collected using PsychoPy 2020.1.1.
Data analysis	Data were analyzed using the following open source software packages: fMRIPrep 21.0.0, freesurfer 6.0.1, nipype 1.6.1, vistasoft 1.0, nilearn 0.9.1, pandas 1.3.3, numpy 1.21.2, scipy 1.10.1, matplotlib 3.4.3, and seaborn 0.11.2. The code used to produce the analyses in this study is available at https://github.com/sfavila/Favila_NatComm_2026 and is also linked to on the OSF page containing the data (https://doi.org/10.17605/OSF.IO/C948A)

For manuscripts utilizing custom algorithms or software that are central to the research but not yet described in published literature, software must be made available to editors and reviewers. We strongly encourage code deposition in a community repository (e.g. GitHub). See the Nature Portfolio guidelines for submitting code & software for further information.

Data

Policy information about availability of data

All manuscripts must include a data availability statement. This statement should provide the following information, where applicable:

- Accession codes, unique identifiers, or web links for publicly available datasets
- A description of any restrictions on data availability
- For clinical datasets or third party data, please ensure that the statement adheres to our policy

The data generated in this study have been deposited on the Open Science Framework (OSF) at https://doi.org/10.17605/OSF.IO/C948A.

Research involving human participants, their data, or biological material

Policy information about studies with human participants or human data. See also policy information about sex, gender (identity/presentation), and sexual orientation and race, ethnicity and racism.

Reporting on sex and gender	Participants self-reported sex and/or gender. Participants could generate any response to this question (no predefined options were provided). Most participants provides biological sex information (male/female), but some also provided gender terms such as "non-binary". These data were collected in order to understand how we are sampling the local population, but were not variables of interest in the study. No analyses on sex/gender were carried out because evaluating sex/gender differences fell outside the primary scope and hypotheses of the study.
Reporting on race, ethnicity, or other socially relevant groupings	Participants self-reported race and ethnicity. Participants could select from six race responses: American Indian/Alaska Native, Asian, Native Hawaiian or Pacific Islander, Black or African-American, White, or Other. They could select more than option and provide their own response if they selected Other. Participants could select from two ethnicity responses: Hispanic or Latino, Not Hispanic or Latino. These data were collected in order to understand how we are sampling the local population, but were not variables of interest in the study.
Population characteristics	See "Research Sample" section below.
Recruitment	Participants were recruited from the Columbia University community through flyers posted on campus and in the nearby community. Because flyers were posted on a university campus with a large number of undergraduates, participants that volunteered for our study were younger, more educated, and were more comfortable with computer-based tasks than the broader young adult population. This enabled us to have shorter experimental sessions that reached high levels of memory performance. We might expect behavioral training to take longer and/or to have more participant attrition in a young adult population without these characteristics.
Ethics oversight	Columbia University Institutional Review Board

Note that full information on the approval of the study protocol must also be provided in the manuscript.

Field-specific reporting

Please select the one below that is the best fit for your research. If you are not sure, read the appropriate sections before making your selection.

☐ Life sciences ☒ Behavioural & social sciences ☐ Ecological, evolutionary & environmental sciences

For a reference copy of the document with all sections, see nature.com/documents/nr-reporting-summary-flat.pdf

Behavioural & social sciences study design

All studies must disclose on these points even when the disclosure is negative.

Study description	Quantitative experimental study
Research sample	A sample of healthy young adults in the local university community was chosen because this study focused on the baseline biological and computational relationships between hippocampal memory representations and visual cortical preparatory activity. Sampling from a young university-affiliated population minimizes confounding age and education-related variations.<br>The final sample of 32 participants (after exclusions) consisted of 15 male individuals, 16 female individuals, and 1 non-binary individual. Participants included 11 individuals who identified as White, 9 who identified as Hispanic/Latino and White, 3 who identified as Black or African-American, 1 who identified as Hispanic/Latino and Black or African-American, 5 who identified as Asian, and 3 who identified as multiracial. Participants were 18–31 years old (median = 21) and had 12–20 years of education (median = 15.5). All participants were right-handed, had normal or corrected-to-normal visual acuity, normal color vision, and no MRI contraindications. This sample is representative for the Columbia University community. All participants provided written informed consent prior to the experiment and were compensated $20/hr for their time. All experimental procedures were approved by the Columbia University Institutional Review Board.
Sampling strategy	Participants were a convenience sample recruited from Columbia University and the surrounding community. We did not use a statistical approach to determine our sample size. We chose our sample size based on previous studies using similar methodology (fMRI studies with within-participant manipulations). Previous studies examining hippocampal representations have detected effects at similar and smaller sample sizes.
Data collection	A computer (Apple Laptop running Psychopy 2020.1.1) , eyetracker (Eyelink 1000 Plus by SR Research), and MRI machine (3T Siemens Prisma) were used to collect data. Only the researchers and undergraduate research assistants certified to interact with human participants were present. Experimental conditions of interest were within-participant, so no blinding to across-participant conditions was required. The experimenters were not blinded to the hypothesis.
Timing	02/2022 - 09/2022
Data exclusions	We excluded all data acquired from four participants from our analyses. Three participants were excluded because they withdrew from the experiment during the first experimental session due to difficulty with the task. MRI data was not acquired for these

participants. One participant was excluded because of low eye-tracking data quality from the MRI session (> 3 standard deviations below the mean number of usable trials across participants). One run of the search task was excluded from one additional participant due to excessive head motion. Exclusion criteria were pre-established.

Non-participation

Three participants withdrew from the experiment during the first experimental session because they found the memory task too difficult.

Randomization

Participants were not allocated to groups. All randomizations (e.g. association between scene stimuli and spatial locations) were performed within participants.

Reporting for specific materials, systems and methods

We require information from authors about some types of materials, experimental systems and methods used in many studies. Here, indicate whether each material, system or method listed is relevant to your study. If you are not sure if a list item applies to your research, read the appropriate section before selecting a response.

Materials & experimental systems

n/a	Involved in the study
☒	☐ Antibodies
☒	☐ Eukaryotic cell lines
☒	☐ Palaeontology and archaeology
☒	☐ Animals and other organisms
☒	☐ Clinical data
☒	☐ Dual use research of concern
☒	☐ Plants

Methods

n/a	Involved in the study
☒	☐ ChIP-seq
☒	☐ Flow cytometry
☐	☒ MRI-based neuroimaging

Plants

Seed stocks

Report on the source of all seed stocks or other plant material used. If applicable, state the seed stock centre and catalogue number. If plant specimens were collected from the field, describe the collection location, date and sampling procedures.

Novel plant genotypes

Describe the methods by which all novel plant genotypes were produced. This includes those generated by transgenic approaches, gene editing, chemical/radiation-based mutagenesis and hybridization. For transgenic lines, describe the transformation method, the number of independent lines analyzed and the generation upon which experiments were performed. For gene-edited lines, describe the editor used, the endogenous sequence targeted for editing, the targeting guide RNA sequence (if applicable) and how the editor was applied.

Authentication

Describe any authentication procedures for each seed stock used or novel genotype generated. Describe any experiments used to assess the effect of a mutation and, where applicable, how potential secondary effects (e.g. second site T-DNA insertions, mosiacism, off-target gene editing) were examined.

Magnetic resonance imaging

Experimental design

Design type

Event-related task

Design specifications

Each participant performed 8 runs of the fMRI search task. Each run contained 56 trials. The first trial in each run was discarded from behavioral analyses since it was not cued. Among the 55 cued trials per run, there were 41 validly cued trials, 7 invalidly cued trials, and 7 no-prediction trials. The 55 cued trials always consisted of two presentations each of the 24 critical (paired) scenes and one presentation each of 7 trial-unique novel scenes. Across all 8 runs of the task, each of the 24 critical scenes was presented 16 times; 12 of these presentations were validly cued trials, 2 were invalidly cued trials, and 2 were no-prediction trials. On each trial, scenes were presented for 1.25 s. At the offset of scene presentation, an intertrial fixation interval began. This interval was randomly selected to be 4.75 s, 6.25 s, or 7.75 s with equal probability.

Behavioral performance measures

Our behavioral performance measures were derived from eyetracking data. We extracted raw gaze data from the scene presentation period of search trials and converted the gaze coordinates from pixels into degrees of visual angle. We removed the 200 ms prior to blinks and the 200 ms after blinks to remove artifacts. We used smoothed gaze data to define saccades as eye movements with a velocity of at least 30 deg/s and an amplitude of at least 0.25 deg. We corrected saccade trajectories based on the longest fixation of the pretrial central fixation period to account for drift. For our analyses, we dropped all saccades that landed within 1 degree of central fixation; of the remaining saccades, we analyzed the end points of the first and last saccade with respect to that trial's distortion location. We statistically compared the proportion of first and last saccades landing within 22.5 degrees of the warp location for each condition of interest (valid, invalid, no prediction). We also considered the precision of the saccades (distances from warp location in degrees) and the proportion of saccades close the competitor location.

Acquisition

Imaging type(s)

functional and structural

Field strength

3T

Sequence & imaging parameters

All MRI data were acquired on a 3T Siemens Magnetom Prisma system located at Columbia University's Zuckerman Mind Brain Behavior Institute. Functional images were acquired using a Siemens 64-channel head/neck coil and a multiband EPI sequence (repetition time = 1.5s, echo time = 30ms, flip angle = 65°, acceleration factor = 3, voxel size = 2 x 2 x 2mm, phase encoding direction = posterior to anterior). Sixty-nine oblique axial slices (14° transverse to coronal) were collected in an interleaved order. Each participant also underwent a T1-weighted anatomical scan (resolution = 1 x 1 x 1mm) using a magnetization-prepared rapid acquisition gradient-echo (MPRAGE) sequence. Double-echo gradient echo images were acquired to estimate the susceptibility-induced distortion present in the functional EPIs. In two participants, an opposite phase encoded field map sequence (anterior to posterior and posterior to anterior) was collected instead.

Area of acquisition

Whole brain

Diffusion MRI ☐ Used ☒ Not used

Preprocessing

Preprocessing software

Preprocessing was performed using fMRIPrep 21.0.0. A B0 nonuniformity map was estimated from the phase-drift map(s) measure with two consecutive GRE acquisitions. The T1-weighted image was corrected for intensity non-uniformity and used as a T1w-reference. The T1w-reference was skull-stripped. Brain tissue segmentation of cerebrospinal fluid, white-matter and gray-matter was performed on the brain-extracted T1w. Brain surfaces were reconstructed using recon-all. For each of the BOLD runs per participant, the following preprocessing was performed. First, a reference volume and its skull-stripped version were generated. Head-motion parameters with respect to the BOLD reference were estimated before any spatiotemporal filtering. The estimated fieldmap was then aligned with rigid-registration to the target EPI reference run. The field coefficients were mapped on to the reference EPI using the transform. BOLD runs were slice-time corrected to 0s for retinotopic mapping runs and 0.708s for memory-guided search runs. The BOLD reference was then co-registered to the T1w reference. Co-registration was configured with six degrees of freedom. The BOLD time series were resampled onto the fsnative surface. All resamplings were performed with a single interpolation step by composing all the pertinent transformations (i.e. head-motion transform matrices, susceptibility distortion correction when available, and co-registrations to anatomical and output spaces). Timeseries were not smoothed.

Normalization

No analyses were performed using data that were normalized. Because this experiment required precise mapping of population receptive fields, which vary in location and size across individuals, all data were analyzed in native participant brain space.

Normalization template

Analyses were not performed on normalized data.

Noise and artifact removal

We implemented motion correction in preprocessing as described above. Additionally, six motion parameters and cosine drift parameters were included as nuisance regressors in our GLMs to control for motion confounds and signal drift. No physiological signals (heartrate, respiration) were recorded.

Volume censoring

No volume censoring was performed.

Statistical modeling & inference

Model type and settings

Mass univariate model (scenes): To estimate the pattern of activity evoked by similar scenes, we conducted a voxelwise GLM analysis of each participant's preprocessed time series data from the search task in native T1 space. Each model contained a regressor of interest for each stimulus in the experiment (24 critical scenes [12 pairs of similar scenes] and 56 novel scenes). Events within these regressors were constructed as boxcars with a duration of 1.25s (scene presentation duration), convolved with a double-gamma hemodynamic response function. Six realignment parameters and cosine drift parameters were included as nuisance regressors to control for motion confounds and signal drift. First level models were estimated using nilearn and contrasts for each of the 24 critical scenes versus baseline were computed. No group-level modeling was performed.

Residuals from mass univariate model (ITI): We examined preparatory BOLD responses during the inter-trial interval period of the search task by first removing the responses evoked by the preceding scene presentation. To remove the visually-evoked responses, we conducted a voxelwise GLM analysis of each participant's preprocessed time series data from the search task in native surface space. Each model contained two regressors of interest: one for presentations of the 24 critical scenes (12 pairs of similar scenes) and one for presentations of the novel scenes. These regressors and nuisance regressors were constructed using the same procedure as described above. No group-level modeling was performed. We took the residual time series after estimating this model in each participant and z-scored each vertex across time and then each time point across vertices.

Encoding model (pRF): We took the preprocessed BOLD time series from the retinotopic mapping task in native surface space and upsampled them to a temporal resolution of 1.0s to match the rate at which the bar stimulus moved. We then averaged them across runs. Using this average time series, we estimated the best fitting pRF for each vertex. Each pRF was modeled as a circular 2D Gaussian, parameterized by values x, y, and σ. The x and y parameters specify the center position of the 2D Gaussian in the visual field. The σ parameter, the standard deviation of the 2D Gaussian, specifies the size of the receptive

	field. Stimulus images from the retinotopic mapping task were converted to contrast apertures. Candidate pRFs were multiplied pointwise by the stimulus apertures to yield a predicted time course of activation by the stimulus, which was then convolved with a hemodynamic response function to predict the BOLD response in each vertex. Using an iterative coarse-to-fine fitting approach, we estimated the x, y, and σ parameters for each vertex that, when applied to the drifting bar stimulus, minimized the difference between the observed and predicted BOLD time series.
Effect(s) tested	Scene Pair Pattern Similarity: To compute scene pair pattern similarity, we calculated the Fisher z-transformed Pearson correlation between the t-maps from the scene GLM for all unique pairwise combinations of the 24 scenes. For each stimulus category (e.g., arches), we averaged all the correlations between nonpairmate scenes that included a scene from that category (e.g., arch 1 and barn 1; arch 2 and barn 1, etc). We subtracted this value from the correlation of the two pairmate scenes from that category (e.g.,arch 1 and arch 2). To assess the overall tendency of a brain region to differentiate similar scenes, we conducted these analyses separately within brain regions of interest and averaged the scene pair pattern similarity values across the 12 scene pairs to produce one value per participant. To test the relationship between hippocampal differentiation and behavior, we sorted the scene pairs by their scene pair pattern similarity value in the hippocampus and divided them into more and less differentiated halves.<br><br>Visual Cortex Preparatory Responses: Using the residual time series from the ITI model, we isolated the time points corresponding to the first 3 TRs of the blank ITI of the search task after accounting for hemodynamic lag. This corresponded to TRs 4, 5, and 6 from scene onset. We averaged the residual time series across these TRs for each trial, generating one ITI BOLD response value per vertex per trial. We organized these responses according to the values of the parameter estimates derived from the pRF model. We first selected the subset of EVC vertices that were potentially responsive to the 8 potential target locations in our experiment. We included vertices whose pRFs met 3 criteria: (1) they had eccentricity values between 0.5 and 10 degrees, indicating they were responsive to the display screen excluding central fixation; (2) the center of the 2D Gaussian was within 2σ of the circle along which target locations were aligned, indicating they were additionally responsive to target and competitor locations; and (3) their pRF model R2 was at least 0.1, indicating they exhibited spatial selectivity. We then binned these vertices into 8 bins according to the polar angle distance between their pRF and the target memory location on the upcoming trial. We z-scored the responses again to ensure that the average response across all bins was zero and then took the median ITI response within a bin before averaging across participants. This procedure produced a spatial response function that indicates how strong the ITI response was in vertices coding for the upcoming target location and how quickly this response falls off over space. Analyses examining competitor location responses during the ITI followed the same procedure but binned vertices according to their distance from the competitor location.

Specify type of analysis: ☐ Whole brain ☒ ROI-based ☐ Both

Anatomical location(s)	The hippocampus was defined bilaterally in each participant's native anatomical space using Freesurfer's automated labeling. Visual cortex regions of interest were defined by visualizing the pRF parameters on each participant's native cortical surface and hand-drawing boundaries between visual field maps. For each of V1, V2, and V3, we drew boundaries at polar angle reversals, following established practice. We combined bilateral V1, V2, and V3 surface masks into a single early visual cortex (EVC) ROI.
Statistic type for inference<br>(See Eklund et al. 2016)	We did not make inferences directly on voxel-wise maps. We aggregrated voxel-wise data into ROI-level summary statistics, as described above.
Correction	No inference was made directly on voxel-wise maps, so no correction was performed.

Models & analysis

n/a	Involved in the study
☒	☐ Functional and/or effective connectivity
☒	☐ Graph analysis
☒	☐ Multivariate modeling or predictive analysis
