# Supplementary material for: Hippocampal mechanisms underlying the resolution of competition in memory and perception
Source: Nat Commun. 2026 Sep 4;17:10528. doi: 10.1038/s41467-026-77400-8 (PMC13639053; doi:10.1038/s41467-026-77400-8)
Supplement: Supplementary file 3 [file 41467_2026_77400_MOESM3_ESM.pdf]

nature portfolio

Peer Review File

Hippocampal mechanisms underlying the resolution of competition in memory and perception

Corresponding Author: Dr Serra Favila

This manuscript has been previously reviewed at another journal. This document only contains information relating to versions considered at Nature Communications.

This file contains all reviewer reports in order by version, followed by all author rebuttals in order by version.

Version 0:

Reviewer comments:

Reviewer #1

(Remarks to the Author)
The authors have done a commendable job improving their work by analyzing regions outside the hippocampus and including additional control analyses. Although I believe this improved manuscript will likely advance our understanding of hippocampal contributions to memory and perception, I still have a few concerns from my previous review that remain unresolved:

1. The authors do not provide enough details regarding their linear mixed-effects models to evaluate whether their statistical inferences are warranted. It appears the authors do not allow slopes to vary by subject in any of their models, which has the potential to inflate Type I errors. In addition, using likelihood ratio tests for inference is also known to be anti-conservative in this context (see Luke 2017, Behavior Research Methods). When performing inference on model parameters, the authors often only report p-values, betas, and standard errors. As far as I can tell, they do not mention what procedure is used to compute p-values nor do they mention how degrees of freedom are estimated. The authors should be clear about their methods, report degrees of freedom and how they were generated using methods that prove adequate Type I error control. The lack of random slopes exacerbates these concerns. As such, I would not have confidence in the reported results relating hippocampal distinctiveness to EVC activity without an appropriate statistical approach.

2. Another concern relates to the interpretation of preparatory activity in EVC. The results and interpretation of this activity as it relates to search success and interference errors (Fig. 4) is straightforward to interpret. However, I believe the authors have oversimplified their interpretation for these effects as they relate to hippocampal pattern similarity (Fig. 5). The authors argue that distinct hippocampal activity for pairmates is associated with the “precision of preparatory representations.” However, the data presented in Figure 5B make it appear as though distinct hippocampal representations are associated with increased non-target activity away from the competitor location. The authors state that “we hypothesized that participants location predictions during the ITI would be represented by spatially-organized activity within these maps.” If one follows this logic, when participants have distinct hippocampal representations they are just as likely to predict a location 180 degrees away from the target. It is hard for me to accept that this equates to a precise representation. While there may be more local precision near the target, when considering the full visual field precision does not increase overall. This point should at the very least be brought up in the discussion, and I think the authors should discuss why such activity does not appear to be relevant for behavior compared to activity at the target and competitor locations.

3. The authors still refer to hippocampal memory differentiation in the abstract and conclusion when they do not quantify hippocampal differentiation in the study. This should be rephrased.

4. The authors frequently omit measures of effect sizes, confidence intervals, and measures of variability when reporting statistical tests. These should be included for proper interpretation of the data.

Reviewer #2

(Remarks to the Author)
I thank the authors for addressing my questions and conducting additional analyses, and I congratulate them on an excellent paper.

Reviewer #4

(Remarks to the Author)
In this interesting study, the authors propose that mechanisms resolving competition in memory also shape selective attention by influencing sensory representations. Using fMRI pattern similarity, receptive field modeling, and eye tracking during a memory-guided visual search task, they show that hippocampal differentiation of competing memories predicts accurate memory-guided eye movements.

I reviewed the authors’ response on behalf of Reviewer 3 and believe that the authors have adequately addressed the reviewer’s comments. I recommend publication of this work and congratulate the authors on this well-conducted study.

I have an additional comment after reading through the manuscript. Because in-scanner eye tracking is both technically challenging and central to the authors’ analyses and conclusions, it would be helpful if the authors could provide quantitative metrics demonstrating the quality and reliability of the eye-tracking data in the present study. For example, reporting measures of data completeness, spatial accuracy/precision, temporal resolution, and calibration stability across runs or participants would increase confidence that the eye-movement results are robust and well supported.

Version 1:

Reviewer comments:

Reviewer #1

(Remarks to the Author)
The authors have done a great job addressing all of my concerns, and I believe this piece makes a valuable contribution to our understanding of hippocampal contributions to memory and perception.

Reviewer #4

(Remarks to the Author)
The authors have addressed all my questions. I do not have further questions.

Open Access This Peer Review File is licensed under a Creative Commons Attribution 4.0 International License, which permits use, sharing, adaptation, distribution and reproduction in any medium or format, as long as you give appropriate credit to the original author(s) and the source, provide a link to the Creative Commons license, and indicate if changes were made.

In cases where reviewers are anonymous, credit should be given to 'Anonymous Referee' and the source.
The images or other third party material in this Peer Review File are included in the article's Creative Commons license, unless indicated otherwise in a credit line to the material. If material is not included in the article's Creative Commons license and your intended use is not permitted by statutory regulation or exceeds the permitted use, you will need to obtain permission directly from the copyright holder.
To view a copy of this license, visit https://creativecommons.org/licenses/by/4.0/

Response Letter for Favila & Aly (NCOMMS-25-45574-T)

Reviewer #1:

The authors have done a commendable job improving their work by analyzing regions outside the hippocampus and including additional control analyses. Although I believe this improved manuscript will likely advance our understanding of hippocampal contributions to memory and perception, I still have a few concerns from my previous review that remain unresolved:

1. The authors do not provide enough details regarding their linear mixed-effects models to evaluate whether their statistical inferences are warranted. It appears the authors do not allow slopes to vary by subject in any of their models, which has the potential to inflate Type I errors. In addition, using likelihood ratio tests for inference is also known to be anti-conservative in this context (see Luke 2017, Behavior Research Methods). When performing inference on model parameters, the authors often only report p-values, betas, and standard errors. As far as I can tell, they do not mention what procedure is used to compute p-values nor do they mention how degrees of freedom are estimated. The authors should be clear about their methods, report degrees of freedom and how they were generated using methods that prove adequate Type I error control. The lack of random slopes exacerbates these concerns. As such, I would not have confidence in the reported results relating hippocampal distinctiveness to EVC activity without an appropriate statistical approach.

Response: We thank the reviewer for raising these important concerns regarding our mixed-effects modeling approach. We have now substantially expanded the Statistics section in the Methods to provide a complete description of the models and the procedures used for statistical inference. We have also adjusted the statistical reporting in the paper in several ways. We would like to highlight three specific adjustments:

Enhanced Statistical Reporting: To provide full transparency, we no longer report only betas and p-values. All results now include degrees of freedom, t-statistics, and 95% confidence intervals.

Adoption of Satterthwaite Approximation: Following the Reviewer's suggestion and the recommendations of Luke (2017), we no longer rely on Likelihood Ratio Tests for inference. We now fit all models using Restricted Maximum Likelihood (REML) and derive degrees of freedom and p-values using the Satterthwaite approximation. As noted by Luke (2017), these approximations produce "acceptable Type 1 error rates even for smaller samples." We utilize this approximation for Type III F-tests when assessing interactions, for estimating 95% CIs for beta coefficients, and for conducting post-hoc comparisons using estimated marginal means.

Random Effects Structure: We recognize that 'maximal' random effects structures are generally preferred to control for Type I error. However, in our specific dataset, models including random slopes sometimes failed to converge or resulted in singular fits, indicating that the complexity of the random effects structure exceeded the information available in the data (Bates et al., 2015).

According to the parsimony principles advocated by Bates et al. (2015), we reduced the complexity of our models in these cases. Nevertheless, to address the reviewer's concern, we have performed a sensitivity analysis to ensure that the results we report are robust. We verified that the effects identified in our parsimonious models remain consistent when compared against the (non-converged) maximal models. Since the Reviewer has a specific concern about the results relating hippocampal distinctiveness to EVC activity, we include a table of our results with the maximal model and reduced model below, according to the conventions described above. This serves as a representative example of the sensitivity analyses we performed across all reported mixed effects models to ensure that our fixed effects conclusions were robust to the choice of random effects structure. As indicated in the Table, the results are similar across the maximal and reduced models, with both models supporting our conclusion (the most critical finding is that target activity ≈ competitor activity for less distinct scene pairs, whereas target activity > competitor activity for more distinct scene pairs).

	**Maximal Model** (not converged)	**Reduced Model**
**Model**	lmer(bold ~ pRF_cond * sep_bin + (pRF_cond + sep_bin | subj), data=pairs45_df, REML=T)	lmer(bold ~ pRF_cond * sep_bin + (1 | subj), data=pairs45_df, REML=T)
**Interaction**	$F_{3,3666}$ = 6.76, p = 0.00015	$F_{3, 19617}$ = 4.78, p = 0.0025
**Less Distinct Pairs**	**Target vs competitor:** β = 0.0011, 95% CI = [-0.015, 0.018], $t_{159.5}$ = 0.13, p = 0.89<br><br>**Target vs control:** β = 0.025, 95% CI = [0.0055, 0.044], $t_{78.7}$ = 2.56, p = 0.0124<br><br>**Competitor vs control:** β = 0.024, 95% CI = [0.0033, 0.044], $t_{66.2}$ = 2.32, p = 0.023	**Target vs competitor:** β = 0.0006, 95% CI = [-0.015, 0.016], t_{19617} = 0.07, p = 0.94<br><br>**Target vs control:** β = 0.023, 95% CI = [0.007, 0.038], t_{19617} = 2.92, p = 0.0035<br><br>**Competitor vs control:** β = 0.022, 95% CI = [0.007, 0.038], t_{19617} = 2.85, p = 0.0044
**More Distinct Pairs**	**Target vs competitor:** β = 0.018, 95% CI = [0.0023, 0.033], $t_{125.4}$ = 2.28, p = 0.024<br><br>**Target vs control:** β = 0.018, 95% CI = [-0.0003, 0.037], $t_{65.0}$ = 1.97, p = 0.053<br><br>**Competitor vs control:** β = 0.0003, 95% CI = [-0.019, 0.020], $t_{55.4}$ = 0.028, p = 0.98	**Target vs competitor:** β = 0.018, 95% CI = [0.004, 0.032], t_{19617} = 2.50, p = 0.013<br><br>**Target vs control:** β = 0.020, 95% CI = [0.005, 0.034], t_{19617} = 2.73, p = 0.0063<br><br>**Competitor vs control:** β = 0.0017, 95% CI = [-0.012, 0.016], t_{19617} = 0.24, p = 0.81

*Note on Degrees of Freedom: While the estimated coefficients and effect directions from each model support the same conclusions, the degrees of freedom differ substantially between models. This is expected: the Satterthwaite approximation penalizes the df in the maximal model to account for the additional uncertainty introduced by estimating subject-specific slopes. Despite this, the critical interaction remains significant.

We report a number of other analyses in the paper establishing the selectivity of this effect to hippocampal scene distinctiveness (i.e. analyses that report no interaction between visual cortex location coding and scene distinctiveness/univariate activity in other ROIs). In all cases, we follow the same procedure: first attempting the maximal model and then removing random slopes until the model converges. In some cases, the data do not support either random slope, and in some cases, they support one of the slopes. We report the results from the most maximal model that converges in the manuscript. However, in all cases we have confirmed that changing the random effects structure of the model does not change the conclusions.

Our revised Statistics subsection in the Methods, which details our approach, is included here:

> "We evaluated the statistical reliability of our behavioral and pattern similarity results using paired t-tests, repeated measures ANOVAs, and Pearson correlations. We report 95% CIs on the difference in means (for t statistics) or for the parameter of interest (for Pearson correlations). We additionally report Cohen's d_z effect size for t statistics and partial eta-squared effect size for repeated measures ANOVAs. Reported p-values are two-tailed and evaluated at alpha = 0.05.
>
> We used linear mixed effects models to assess the link between preparatory responses in visual cortex and 1) behavior; 2) hippocampal distinctiveness. For all models described below, we initially fit a model including the maximal random effects structure (Barr et al., 2013). However, in cases of non-convergence or singular fits, which indicate that the random effect structure is overparameterized for the complexity of the data, we removed random slopes until convergence was achieved. We always included a random intercept for each participant. We therefore report results from the most parsimonious model that converged and yielded stable parameter estimates (Bates et al., 2018). We note that all significant effects identified in parsimonious (random intercept-only) models remain significant and consistent in direction when compared against the maximal models. Following best practices to reduce Type 1 error rate, we fit all models with Restricted Maximum Likelihood and used the Satterthwaite approximation to calculate degrees of freedom and report p values (Luke, 2017). All models were implemented in the lme4 (Bates et al., 2015), lmerTest (Kuznetsova et al., 2017), and emmeans (Lenth & Piaskowski, 2026) packages for R.
>
> To examine the relationship between preparatory responses in visual cortex and behavior on the subsequent trial, we fit linear mixed effects models to single trial data. For each valid trial and participant, the ITI BOLD response was extracted from the eight pRF bins described above and entered as the dependent variable. Trials in which no eye movement response

was made on the subsequent trial were excluded from analysis. Behavioral outcome on the subsequent trial ("success", "interference", or "other") and pRF location labels ("target", "competitor", "control", or "other") were included as fixed effects, along with their interaction. The most parsimonious model had the following specification: lmer(bold ~ pRF_cond * behav_outcome + (1 | subj), data=valid_trials, REML=T). The pRF location x behavioral outcome interaction was first assessed using Type III F-tests. To probe the interaction, estimated marginal means were used to perform planned pairwise comparisons between pRF locations within each behavioral outcome. For each comparison, we report the estimated coefficient (β), 95% CI, t statistic, degrees of freedom, and p value.

To examine whether hippocampal memory representations predicted preparatory activity in visual cortex for 45 degree pairs, we fit a linear mixed-effects model relating hippocampal distinctiveness to ITI responses in different pRF voxel groups. As in the prior analysis, the ITI BOLD response extracted from each of the eight pRF bins for each valid trial and participant served as the dependent variable. Hippocampal distinctiveness was computed as pattern similarity at the level of each scene pair (see *Methods/Pattern Similarity Analyses*) and sorted in low distinctiveness (high pattern similarity) and high distinctiveness (low pattern similarity) groups. Hippocampal distinctiveness ("low", "high") and pRF location labels ("target", "competitor", "control", or "other") were included as fixed effects, along with their interaction. The most parsimonious model had the following specification: lmer(bold ~ pRF_cond * sep_bin + (1 | subj), data=pairs45_data, REML=T). As before, the pRF location x hippocampal distinctiveness interaction was first assessed using Type III F-tests from the mixed effects model. To probe the interaction, estimated marginal means were used to perform planned pairwise comparisons between pRF locations within each hippocampal distinctiveness group. For each comparison, we report the estimated coefficient (β), 95% CI, t statistic, degrees of freedom, and p value.

To examine whether these effects generalized across pair distance, we fit additional models that tested 90 degree, 135 degree, and 180 degree pairs using the same fixed effects model structure. We also tested the three-way interaction between hippocampal distinctiveness, pRF location, and target-competitor distance. We used a similar mixed-effects modeling approach described above; however, for these analyses, control pRFs were omitted because some pair distances (e.g., 180°) did not allow for a valid definition of a control location, and including them would have resulted in missing cells and non-estimable parameters in the F test. This had no bearing on our primary effect of interest, which was in the relative activity of voxels coding for target vs. competitor locations. In addition, to assess the anatomical specificity of the distinctiveness effect, we fit additional models using data from ROIs besides the hippocampus. Finally, we examined univariate activity instead of our pattern similarity-derived distinctiveness measure. We tested the interaction between pRF location and univariate activity in the DAN using the same modeling framework. Univariate activity was estimated from the same GLM used for the pattern similarity analyses (see *Methods/Pattern Similarity*

Analyses), with t statistics for each scene averaged over voxels in the DAN to provide a measure of univariate activity for each scene pair."

2. Another concern relates to the interpretation of preparatory activity in EVC. The results and interpretation of this activity as it relates to search success and interference errors (Fig. 4) is straightforward to interpret. However, I believe the authors have oversimplified their interpretation for these effects as they relate to hippocampal pattern similarity (Fig. 5). The authors argue that distinct hippocampal activity for pairmates is associated with the "precision of preparatory representations." However, the data presented in Figure 5B make it appear as though distinct hippocampal representations are associated with increased non-target activity away from the competitor location. The authors state that "we hypothesized that participants location predictions during the ITI would be represented by spatially-organized activity within these maps." If one follows this logic, when participants have distinct hippocampal representations they are just as likely to predict a location 180 degrees away from the target. It is hard for me to accept that this equates to a precise representation. While there may be more local precision near the target, when considering the full visual field precision does not increase overall. This point should at the very least be brought up in the discussion, and I think the authors should discuss why such activity does not appear to be relevant for behavior compared to activity at the target and competitor locations.

Response: We thank the reviewer for this thoughtful comment regarding the interpretation of preparatory activity in early visual cortex (EVC) and its relationship to hippocampal pattern similarity. We agree that considering the full polar angle reconstruction raises an important question about how to interpret activity at locations distant from the target, including the apparent signal at 180° for scene pairs with target-competitor distance of 45° (Figure 5B).

To better understand this effect, we conducted additional analyses examining the spatial profile of EVC responses as a function of pRF properties. These analyses revealed that, for scene pairs that were more distinct in hippocampus, the activity at 180° from the target is primarily driven by voxels in our analysis that have spatial receptive fields with low eccentricity estimates (i.e. close to the fovea)[1]. When examining these low eccentricity voxels separately, we observed that there is a very weak/absent response to the target location, as well as an *elevated* response to the location 180° from the target (below, left). However, when we examined voxels with higher eccentricities, a more expected pattern emerges: an elevated response to the target location and no response to the location 180° from the target (below, middle and right). Note that in all cases, we are only including voxels whose pRF size indicates that they are responsive to the possible distortion locations.

[1] Note that all voxels with eccentricities < 0.5 degrees and all voxels with pRF model $R^2 < 0.1$ are excluded from analysis. Voxels that meet these criteria are included in our analyses if their receptive field size is large enough to encompass the possible distortion locations. Thus, low eccentricity voxels would be included only if they have large enough pRFs to be responsive to the distortion.

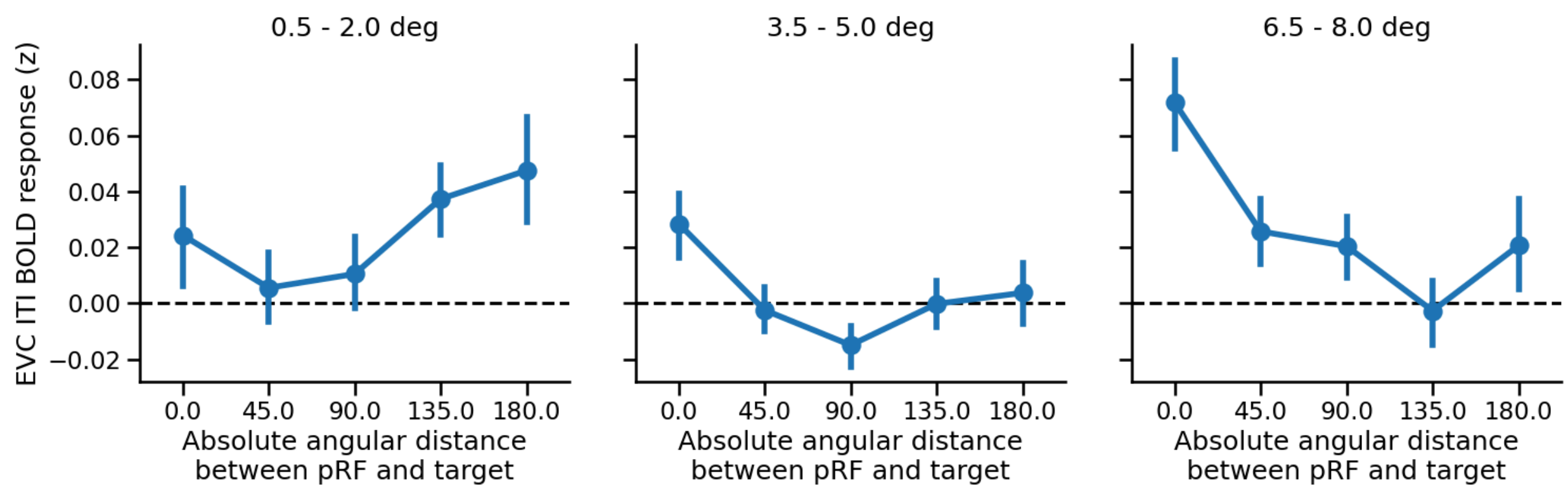

The pattern we observed in the low eccentricity voxels could emerge due to errors in pRF estimation: near the fovea, small positional differences in the visual field can correspond to large changes in polar angle (see below). In addition, our pRF mapping protocol relied on bar apertures rather than wedge/ring stimuli. While bar stimuli are overall the most efficient and provide robust estimates of pRF position and size, they are less optimal for the fovea specifically. This is because bar stimuli simultaneously stimulate many polar angles within a small region of space at the fovea. This choice may have further reduced the precision with which polar angle could be estimated for low eccentricity voxels in our experiment. The fact that these voxels do not exhibit robust responses to the target location is consistent with the interpretation that errors in pRF estimation explain the response.

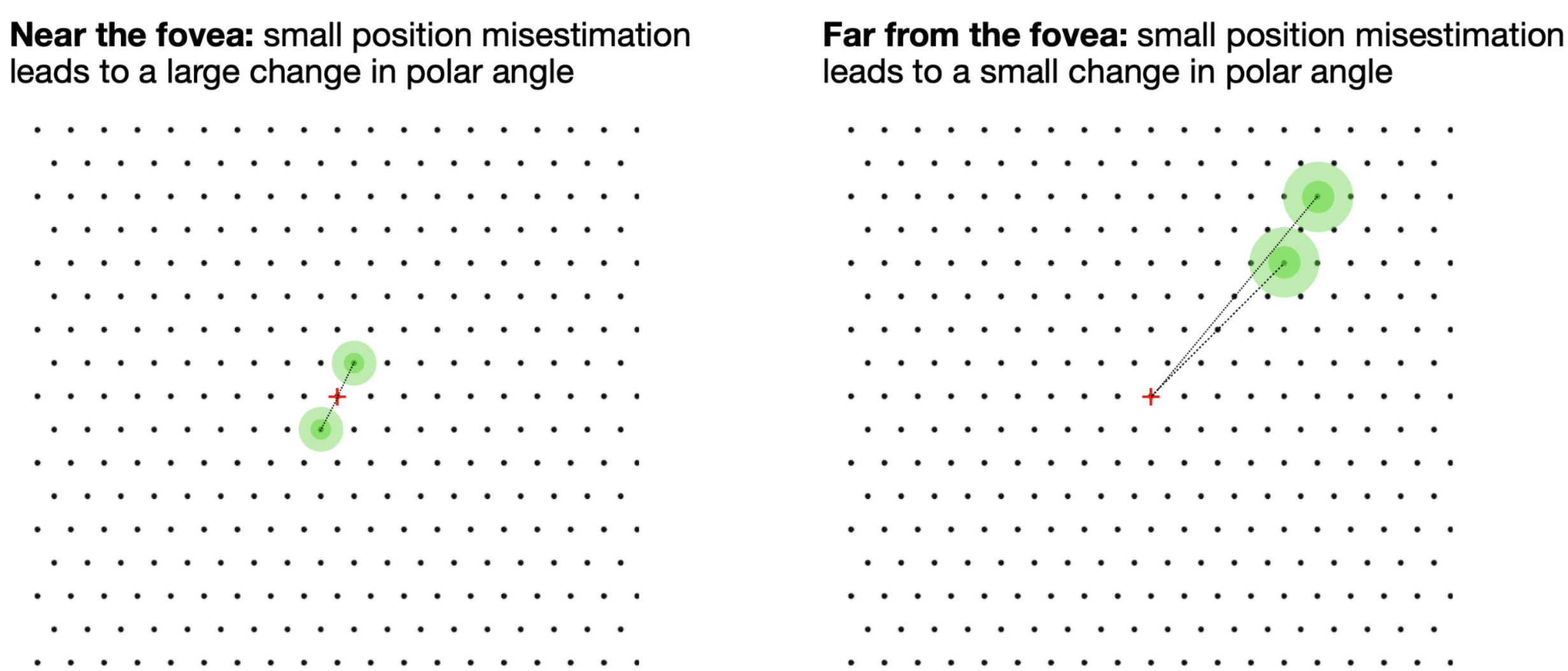

These analyses identify the voxels that drive the signal at 180° and indicate that errors in pRF estimation near the fovea could be an issue; however, this does not by itself explain the observed effect. Specifically, it is unclear why errors in pRF estimation would only affect the observed response for scene pairs that are more distinct in hippocampus. In the Discussion, we now note this elevated signal at 180°, describe the voxels that are its source, confirm that removing those low-eccentricity voxels reduces the signal at 180° but leaves our effects of interest intact, and point out that eye movements are rarely made to that location – suggesting that this signal does

not drive behavior toward the location orthogonal to the target. We also speculate that this effect could be related to the finding that unprioritized items in working memory can be remapped into a different portion of representational space than prioritized items, potentially to decrease interference (Yu et al. 2020). Future studies can test whether the enhanced 180° signal that we observed could reflect a remapped representation of the competitor, with this remapping serving to reduce interference errors by driving apart the representations of the target and competitor in visual cortex. The specificity of this finding to scene pairs that were more distinct in hippocampus points to the possibility that hippocampal mechanisms might contribute to remapped representations in visual cortex, an intriguing idea that requires further investigation.

The added paragraph in the Discussion is included below:

> "While our primary analyses focused on target, competitor, and control locations, we also observed an elevated preparatory signal 180 degrees from the target location for scene pairs that were more distinctive in the hippocampus (Figure 5B, right). Exploratory analyses revealed that this signal was primarily driven by early visual cortex voxels with low eccentricity pRFs. Participants rarely made eye movements toward the 180 degree location (SFig. 2A), suggesting that the signal arising from these voxels during the ITI is unlikely to correspond to overt behavior in the upcoming search task. Importantly, when we removed voxels with low eccentricity pRFs from our analysis, it reduced the 180 degree signal, but our primary interaction between hippocampal scene distinctiveness and pRF (target, competitor, control, and other) locations remained intact (Type III F-test: $F_{3, 19617} = 3.73$, $p = 0.011$). This 180 degree signal may partially be explained by errors in pRF estimation near the fovea, where small positional differences in estimated pRF centers can lead to large differences in the polar angle estimate. However, this would not explain why the signal is only present for scenes pairs that were more distinctive in the hippocampus. One speculative possibility is that some of this signal reflects a form of representational remapping, similar to what has been observed in working memory studies. In these studies, unprioritized items are remapped to a different portion of the representational space in order to decrease competition with prioritized items (Yu et al., 2020). In this framework, the enhanced signal at 180 degrees may minimize interference by maximizing the distance between the target and competitor in visual cortex. The specificity of this finding to scene pairs that were more distinct in the hippocampus points to the possibility that hippocampal mechanisms might contribute to remapped representations in visual cortex, an intriguing idea that requires further investigation."

3. The authors still refer to hippocampal memory differentiation in the abstract and conclusion when they do not quantify hippocampal differentiation in the study. This should be rephrased.

Response: Thank you for pointing out this oversight. We have adjusted language in the Abstract and Discussion sections. The revised Abstract is below:

> "Behaving adaptively requires selection of relevant memories and sensations and suppression of competing ones. We hypothesized that these mechanisms are linked, such that hippocampal computations that resolve competition in memory also shape the precision of sensory representations to guide selective attention. We leveraged fMRI-based pattern similarity, receptive field modeling, and eye tracking to test this hypothesis in humans performing a memory-dependent visual search task. In the hippocampus, more distinct representations of competing memories predicted the accuracy of memory-guided eye movements. In visual cortex, preparatory coding of remembered target locations predicted search successes, whereas preparatory coding of competing locations predicted search failures due to interference. These effects were linked: more distinct hippocampal memories were associated with lower competitor activation in visual cortex, yielding more precise preparatory representations. These results demonstrate a role for memory distinctiveness in shaping the precision of sensory representations, highlighting links between mechanisms that overcome competition in memory and perception."

We made similar edits in the Discussion, including in the concluding paragraph:

> "To conclude, we show that hippocampal mechanisms for distinguishing competing memories are associated with more accurate attentional selection and with more precise preparatory activity in the visual system. Our results demonstrate linked mechanisms that overcome competition in memory and perception and argue for a broad role for memory systems in shaping adaptive perceptual behavior."

4. The authors frequently omit measures of effect sizes, confidence intervals, and measures of variability when reporting statistical tests. These should be included for proper interpretation of the data.

Response: We have revised statistical reporting in the paper. For ANOVAs, we now report partial eta-squared effect size in addition to the F statistic and p values previously reported. For t-tests (including follow-up tests to an ANOVA), we now report the difference in means, the 95% CI on this difference, and Cohen's d_z effect size in addition to the t statistic and p values previously reported. For ease of reading, we do not report individual condition means and standard errors because these are clearly visualized in our figures and because we already report the difference in means.

As an example, here is one paragraph from our revised Results section:

> "To test whether participants were using memory to improve search performance, we examined the accuracy of participants' saccades across the three conditions. Similar to standard attentional cueing effects, we expected participants to perform best in the valid condition, when their predictions were accurate, and worst in the invalid condition, when their

predictions were systematically wrong. To verify this, we calculated the angular error between participants' first saccade end point and the center of the distortion location on each trial and scored this saccade as accurate if it was closer to the distortion than to any of the other 7 possible locations (<22.5 degrees of angular error). We expected the first saccade of the search interval to show the strongest evidence for a memory-based strategy if participants were using the previous scene cue to prepare a saccade. As predicted, we found highly reliable differences in accuracy across the three conditions (valid = 45.4%; no-prediction = 21.5%; invalid = 16.9%; $F_{2,62} = 78.7$, $p < 0.001$; $\eta_p^2 = 0.72$; Fig. 2C). Participants were more likely to make accurate first saccades on valid trials compared to no-prediction trials (valid - no-prediction = 0.24, 95% CI = [0.18, 0.30]; $t_{31} = 8.72$, $p < 0.001$; $d_z = 2.30$) and invalid trials (valid - invalid = 0.28, 95% CI = [0.23, 0.34]; $t_{31} = 9.74$, $p < 0.001$; $d_z = 2.91$). Participants were also less likely to make accurate first saccades on invalid trials than on no-prediction trials (invalid - no-prediction = -0.04; 95% CI = [-0.07, -0.02]; $t_{31} = -3.44$, $p = 0.0016$; $d_z = 0.60$). These effects are consistent with previously reported attentional cueing effects, including memory-based attentional cueing (Summerfield et al., 2006); they verify that participants prepared a saccade to the target location, yielding performance benefits when the distortion appeared there and performance decrements when it unexpectedly appeared elsewhere. We next examined the final saccade of the search interval to test whether these effects persisted into the trial. Relative to the first saccade, the final saccade of the search interval was more accurate in every condition (Supplementary Fig. 1A). However, condition differences were still highly significant for the final saccade ($F_{2,62} = 65.9$, $p < 0.001$; $\eta_p^2 = 0.68$), with valid performance highest and invalid performance lowest (all pairwise comparisons significant at $p < 0.001$ and $d_z > 0.61$; Supplementary Fig. 1A). These results establish that participants were using memory for the scene-locations associations to guide visual search."

Reviewer #2:

I thank the authors for addressing my questions and conducting additional analyses, and I congratulate them on an excellent paper.

Response: Thank you for your thoughtful comments on the paper and for reviewing our revisions.

Reviewer #4:

In this interesting study, the authors propose that mechanisms resolving competition in memory also shape selective attention by influencing sensory representations. Using fMRI pattern similarity, receptive field modeling, and eye tracking during a memory-guided visual search task, they show that hippocampal differentiation of competing memories predicts accurate memory-guided eye movements.

I reviewed the authors' response on behalf of Reviewer 3 and believe that the authors have adequately addressed the reviewer's comments. I recommend publication of this work and congratulate the authors on this well-conducted study.

I have an additional comment after reading through the manuscript. Because in-scanner eye tracking is both technically challenging and central to the authors' analyses and conclusions, it would be helpful if the authors could provide quantitative metrics demonstrating the quality and reliability of the eye-tracking data in the present study. For example, reporting measures of data completeness, spatial accuracy/precision, temporal resolution, and calibration stability across runs or participants would increase confidence that the eye-movement results are robust and well supported.

Signed review from Shuo Wang, Washington University in St. Louis

Response: Thank you for assessing our revisions. We agree that reporting quantitative measures of eye-tracking quality strengthens the manuscript. We have added several additional methods details and metrics from our preprocessed gaze data, which we now report in the Methods/Eye Tracking Data Acquisition section:

> "Re-calibration was performed when the experimenters observed high levels of noise in tracked gaze position during fixation periods, when gaze tracking was lost intermittently during the search interval, or when validation tests failed."

And the Methods/Eye Tracking Preprocessing section:

> "To assess the reliability of eye-tracking data collected in the scanner, we computed several data quality measures on the preprocessed gaze estimates from the search task. Calibration was performed on 43% of runs, with wide variability between participants (min = 12.5%, max = 100%), depending on experimenter assessment of eye-tracking quality and the results of validation tests at the start of every run. The mean final calibration error (the average deviation between the measured and intended target positions during the final calibration) was 0.47 degrees of visual angle (SD = 0.27 degrees of visual angle). After preprocessing (including blink removal), data completeness during the critical search period was high across participants (mean valid samples = 88.6%, SD = 6.1%). Because gaze position changed rapidly during the search period, we quantified spatial precision from the ITI fixation period, when participants maintained a stable gaze. For each run, the standard deviation of x- and y-coordinate gaze samples was computed, and spatial precision was defined as the mean of these two values. Spatial precision during this period was 0.46 degrees of visual angle (SD = 0.18 degrees of visual angle), indicating low sample-to-sample variability and good measurement quality."

Note that the sampling rate (1000 Hz) is already reported in the section on Eye Tracking Data Acquisition. Taken together, these metrics indicate that the in-scanner eye-tracking data were of high quality and support the robustness of our memory-guided eye movement results.
